# Effects of self-management education programmes on self-efficacy for osteoarthritis of the knee: a systematic review of randomised controlled trials

**DOI:** 10.1186/s12891-021-04399-y

**Published:** 2021-06-05

**Authors:** Daisuke Uritani, Hitoshi Koda, Sho Sugita

**Affiliations:** 1 Department of Physical Therapy, Faculty of Health Science, Kio University, 4-2-2, Umaminaka, Koryocho, Kitakatsuragigun, 6350832 Nara, Japan; 2 Department of Rehabilitation Sciences, Faculty of Allied Health Sciences, Kansai University of Welfare Sciences, 3-11-1 Asahigaoka, Kashihara city 5820026 Osaka, Japan; 3 Luxem Co., Ltd, 1-10-1 Higashiikuta, Tama-ku, Kawasaki city 2140031 Kanagawa, Japan

**Keywords:** Patient education, Osteoarthritis, Knee, Pain, Self-efficacy

## Abstract

**Background:**

Enhancing self-efficacy to manage symptoms and functions is an important aspect of self-management for patients with knee osteoarthritis (OA). Many reports have investigated the effects of self-management education programmes for arthritis patients. However, a study that exclusively focuses on patients with OA in the same joints is required to clarify the effects of self-management programmes because individuals with knee OA experience physical and psychological difficulties different from those experienced by individuals with other arthritis diseases. Furthermore, previous studies have reported a wide range of delivery styles of self-management education programmes. This systematic review aimed to evaluate the effects of group-based and face-to-face self-management education programmes conducted by health professionals targeting self-efficacy for knee OA exclusively.

**Methods:**

The MEDLINE, CENTRAL, EMBASE, CINAHL, Web of Science, and PEDro databases were searched to identify quantitative measures used in randomised controlled trials (RCTs) to assess the effects of self-management education programmes targeting self-efficacy in patients with knee OA. We included studies in which medical professional-delivered self-management education programmes were conducted in a group-based and face-to-face manner in community or outpatient settings.

**Results:**

Seven RCTs from five countries were included in this review. Our retrieved studies included various types of self-management education programmes such as cognitive behavioural counselling, pain management education, physical education, weight management education, and arthritis self-efficacy management education, and control arms. They assessed various aspects of self-efficacy, including pain, physical function, arthritis symptoms excluding pain, weight management, mobility, and self-regulation. The total score of the Arthritis Self-Efficacy Scale was also measured. Some studies have reported beneficial effects of group-based and face-to-face self-management education programmes on self-efficacy for management of pain and other symptoms and for self-regulatory, knee OA. However, the results of the included studies were varied and inconsistent.

**Conclusions:**

The current review only included seven studies, and there was a wide range of clinical heterogeneity among these studies. Thus, the effects of group-based and face-to-face self-management education programmes conducted by health professionals on self-efficacy for knee OA exclusively are inconclusive to date. Therefore, high-quality studies are required to provide significant information on clinicians, patients, and healthcare professionals in the future.

**Supplementary Information:**

The online version contains supplementary material available at 10.1186/s12891-021-04399-y.

## Background

Knee osteoarthritis (OA) is a common and costly chronic condition that leads to physical symptoms and functional limitations in the elderly [1, 2] and has a negative impact on their quality of life [3]. The Osteoarthritis Research Society International (OARSI) guideline lists core treatments, including land-based exercise, weight management, strength training, water-based exercise, and self-management and education, for the management of patients with knee OA [4]. Exercise is one of the most important approaches to improve pain, function, and quality of life [5]. The OARSI guideline adds that self-management and education are equally indispensable for the core treatment of all individuals [4, 6].

Barlow et al. [7] defined self-management as “the individual’s ability to manage the symptoms, physical treatment, psychological consequences, and lifestyle changes inherent in living with a chronic condition”. The ability to self-manage is usually achieved through patient education programmes (i.e., self-management education). Self-management education programmes include different types of programmes, such as education on disease pathology and progression, cognitive behavioural therapy, and pain coping skills training. One meta-analysis demonstrated that the presence of a psychological component particularly boosted the effectiveness of self-management courses for certain outcomes [8].

Self-management education programmes develop stronger self-beliefs among patients in terms of their abilities, especially their ability to manage symptoms and function also known as self-efficacy [9–12]. Self-efficacy plays an important role in the development of pain coping skills [13] and improves mobility performance [14]. It is also an important determinant of healthy behaviour and encourages the adoption of healthy life activities when suffering from chronic illnesses [9, 10, 15]. A systematic review of patient education identified self-efficacy as a key mediator of behavioural change [15]. Patients with chronic illnesses find it challenging to change their daily behaviour and maintain it [16]; for example, people with knee OA lack self-efficacy because they believe that little can be done to alleviate the impact of OA [15]. Therefore, enhancing self-efficacy to manage symptoms and functions is an important aspect of self-management for knee OA patients [9, 10].

The effectiveness of patient education on self-efficacy for arthritis patients has been reported by several studies [17–26]; however, the evidence available is inconclusive. Most studies included not only individuals with knee OA but also those with other arthritis diseases, such as hip OA and rheumatoid arthritis (RA) [17–26]. This may be one of the reasons why the evidence remains inconclusive. We considered that individuals with knee OA experienced physical and psychological difficulties different from those experienced by individuals with other arthritis diseases; one reason for this is the difference in pathological characteristics. For example, factors associated with physical activities are different between individuals with knee OA and those with hip OA [27, 28]. Therefore, patient education should be tailored differently to cater to different diseases. In the systematic review regarding self-management intervention benefits, Devos-Comby et al. [15] described the need for studies designed for patients experiencing OA exclusively and in the same joints to clarify the effect of self-management programmes.

Furthermore, group-based patient education programmes are more effective than one-to-one programmes [8]. Previous studies demonstrated that exercising with others offered more physical and mental benefits than exercising alone [29–31]. An economic report on chronic disease self-management programmes suggested that group-based self-management interventions were more cost-effective than individual ones [32].

Effects of self-management education on self-efficacy for arthritis patients were evaluated in a systematic review/meta-analysis [33]. This review included studies that analysed patient education programmes conducted by laypeople or non-medical practitioners [33]. However, another systematic review indicated that group-delivered self-management intervention programmes with psychological components led by health care professionals were more beneficial than programmes delivered by laypeople [8].

As mentioned previously, the effects of self-management education on knee OA exclusively have been inconclusive. This may be because the previous similar reviews included a wide range of arthritis subjects and delivery styles. Therefore, this systematic review aimed to evaluate the effect of group-based and face-to-face self-management education programmes conducted by health professionals on self-efficacy in individuals with knee OA exclusively.

### Methods

The protocol for this systematic review was registered with the international prospective register of systematic reviews-PROSPERO (Registration # CRD42018112067).

### Eligibility criteria

#### Study designs

We searched for RCTs that investigated the effectiveness of group-based and face-to-face self-management education programmes conducted by health professionals in people with knee OA. Studies published in peer-review journals were included in this systematic review. We excluded conference abstracts, trial register information, and book chapters. Articles that were not research articles, such as letters, editorials, comments, opinions, and correspondence, were also excluded.

#### Population

 We included studies that recruited participants who were 18 years or older with knee OA. In the present review, we only included tibiofemoral OA because we believe that there was clinical heterogeneity between patellofemoral and tibiofemoral OA in terms of pathology, patient complaints, and rehabilitation. Furthermore, we included studies that explicitly stated that the participants included in the study were diagnosed with knee OA by a medical doctor or met the diagnostic criteria for knee OA defined by any clinical practice guidelines. Studies that included study participants with other types of arthritis, such as hip OA or RA, were excluded if data on knee OA were not presented separately.

#### Intervention

We included studies with group-based interventions and face-to-face patient education programmes designed to enhance self-management capabilities. A self-management education programme is defined as a programme focusing on education about knee OA, OA self-management or self-care, and pain coping skills, as well as self-management of diet [7, 34]. Studies that included intervention programmes other than self-management education programmes (e.g., only home exercise programmes, only given an instructional brochure) were excluded. Interventions conducted remotely through telephones, internet, and letters were excluded. Interventions conducted by non-clinical professionals were also excluded.

#### Comparator

The acceptable comparators were no intervention controls and comparison groups including interventions other than self-management education programmes.

#### Outcome of interest

The outcome of interest was self-efficacy, measured with any kind of self-efficacy scale.

#### Setting

We included studies with intervention programmes in an outpatient/community setting and excluded those in an in-patient care/homebound setting.

### Information sources and search strategy

This review was conducted and reported in line with the Preferred Reporting Items for Systematic Reviews and Meta-Analysis (PRISMA) statement and satisfied the PRISMA checklist [35]. The following databases were searched from inception to the 18th of November 2020: MEDLINE, Cochrane Central Register of Controlled Trials (CENTRAL), EMBASE, Cumulative Index to Nursing and Allied Health Literature (CINAHL), Web of Science, and PEDro. Literature search strategies were developed using Medical subject headings (MeSH) terms and the text words related to knee OA and patient education. Searches were limited to literature written in English or Japanese. Further studies were sought from previous systematic reviews/meta-analyses and literature reviews by manual search. The detailed search strategy for MEDLINE is presented in Table 1. We customised the search strategies according to the search function of each database, such as MeSH terms and truncation.

**Table 1:** Search terms for the systematic review of the literature in MEDLINE

#1	“Knee” [MeSH Terms] OR “knee” [All Fields] OR “knee joint“[MeSH Terms] OR “knee joint“[All Fields]
#2	“Osteoarthritis” [MeSH Terms] OR “osteoarthritis” [All Fields] OR “pain” [MeSH Terms] OR “pain” [All Fields]
#3	#1 AND #2
#4	“Patient Education as Topic” [MeSH Terms]
#5	(“patients” [MeSH Terms] OR “patients” [All Fields] OR “patient” [All Fields]) AND (“education” [MeSH Terms] OR “education” [All Fields])
#6	“health education“[MeSH Terms] OR “health education“[All Fields] OR ((“health” [MeSH Terms] OR “health” [All Fields]) AND (“education” [MeSH Terms] OR “education” [All Fields]))
#7	#4 OR #5 OR #6
#8	“randomized controlled trial“[Publication Type] OR “randomized controlled trial“[TIAB] OR “randomised controlled trial“[TIAB] OR “RCT”[TIAB]
#9	#3 AND #7 AND #8

### Data management

#### Study selection and data management

Search results from each electronic database were transported into EndNote X8 (Thompson Reuters, Carlsbad, California, USA) to organise and sort the identified studies. After deleting duplicates, titles and abstracts were independently screened with reference to the eligibility criteria mentioned above. Two authors (DU and HK) managed literature lists using Microsoft Excel spreadsheets. Decisions were made as to whether to retrieve the full text. In case of a disagreement between the two authors (DU and HK), the decision to retrieve the full text was taken through consensus after a discussion. We obtained the full texts of articles that appeared to possibly satisfy the inclusion criteria. Then, two study authors (DU and HK) independently read the full-text articles and decided on the final list of eligible studies. A third reviewer (SS) was involved in resolving disagreements in each screening phase, when necessary. The excluded studies and reasons for excluding them were recorded.

#### Assessment of the risk of bias of the selected studies

Two study authors (DU and HK) independently assessed the quality of the included studies using the Cochrane Risk of Bias tool [36]. The reviewers rated the selection bias, performance bias, detection bias, attrition bias, and reporting bias of the studies and classified them into ‘low risk’, ‘high risk’, and ‘unclear risk’ categories. Disagreements between the reviewers were resolved through discussion. A third reviewer (SS) was also involved in resolving disagreements in each screening phase, when necessary.

#### Data extraction

One reviewer (DU) extracted information from the studies. Two other reviewers (HK and SS) verified the extracted data. We extracted the following data from each article:

- Publication details (author and year).
- Study details (sample size and inclusion criteria of participants).
- Participant details (age, sex, symptom duration, level of education).
- Intervention details (description, number of participants per session, volume, programme instructors/facilitators, follow-up periods)
- Outcome measures.

For the synthesis of results, we computed effect sizes using standardised mean differences. If a particular study did not report complete data, including mean and standard deviation at each follow-up time point, we e-mailed the authors with a request to provide the missing data. A second e-mail was sent to the study authors after about 1 or 2 weeks as a final reminder if they did not respond to the first.

## Results

### Study selection

**Fig. 1:**
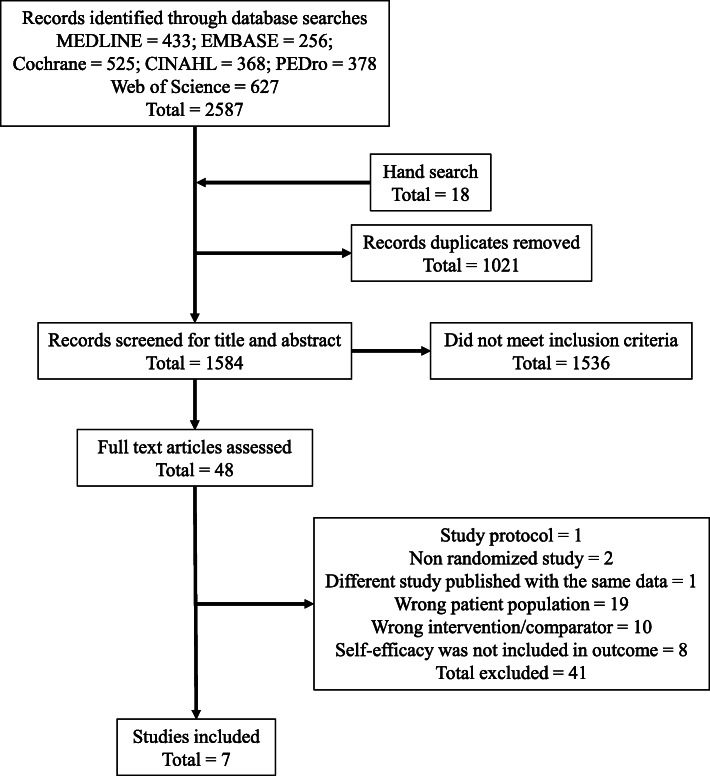
Flow diagram of article identification

Search results are summarised, and a flow diagram of the study design is presented in Fig. 1. The literature search returned 2587 results. In hand search, we checked the references of review articles from the articles included for title screening. We added 18 articles that were not included in the 2587 results for title screening. Of the total 2605 studies, 1021 duplicates were removed. Titles and abstracts of 1584 articles were screened, and 1536 irrelevant articles were excluded based on the eligibility criteria. The full texts of the 48 studies were reviewed, and 41 of the 48 studies were excluded. Nineteen studies were excluded due to incorrect patient population, 10 due to incorrect intervention/comparator, eight because self-efficacy was not included in the outcome, and two due to a non-randomised nature. One article was excluded because it was study protocol, and one because a different study was published using the same data. There was a possibility that two of the seven remaining studies used the same procedures and included the same population [37, 38]. We contacted the authors of these studies for confirmation; however, we did not receive their response. We did not have clear criteria for excluding them and could not determine if these studies were duplicates. Therefore, we included both studies in this review. Finally, seven articles met the eligibility criteria and were assessed for risk of bias.

### Study characteristics

Additional file 1 shows the characteristics of the included studies. Study participants were recruited in US [39, 40], Malaysia [41], France [42], Finland [43], and Hong Kong [37, 38]. To include study participants, four studies confirmed radiographic knee OA [39–41, 43], and three studies used American College of Rheumatology clinical criteria [37, 38, 42]. Sample sizes in the selected studies ranged between 80 [39] and 300 [41]. All studies included knee OA patients exclusively. Participants in one study were overweight and obese patients with knee OA [40]. The mean age of participants in the included studies ranged from 58 [40] to 66.6 years [42]. Most participants in the included studies were female. Self-efficacy for pain was assessed in five studies [37, 38, 41–43], that for physical function was assessed in one study [42], and that for other symptoms was assessed in three studies [37, 38, 42]. The total score of the Arthritis Self-Efficacy Scale (ASES), which included self-efficacy scores for pain, physical function, and other symptoms, was measured in one study [40]. Self-regulatory self-efficacy and self-efficacy for mobility [39] and weight management [40] were measured in two studies, respectively.

### Type of interventions

Details of interventions followed in each of the included studies are provided in Additional file 2. Various education programmes were used in the included studies, such as group-based cognitive behavioural counselling [39, 41], pain management education [40, 43], physical education on the importance of physical exercise and its integration in daily life [42], weight management education [40], and arthritis self-efficacy management education [37, 38]. Five studies included exercise programmes in addition to the education programme [37–40, 42]. The frequencies and durations of interventions varied from three sessions over 6 weeks [41] to 27 sessions over 36 weeks [39].

### Type of comparison arms

Provided programme for comparison arms of interventions in each of the included studies are shown in Additional file 2. Two studies provided conventional orthopaedic intervention [38, 39], and the other two studies provided standard care (including education booklet) [41, 42]. Exercise programme [40], spa therapy and education booklet [43], and ordinary general practioner’s (GP) care were also provided. In six of seven included studies, the intervention arm’s programme consisted of a comparison arm’s programme and a self-management education programme [37–41, 43].

### Assessment of the risk of bias in the selected studies

Qualities of the studies are summarised in Table 2. Three studies were categorised as ‘low risk’ for most of the bias risks [39, 42, 43]. Six of the seven studies [37–42] were categorised as ‘high risk’ for performance bias, as blinding of the personnel and patients were not attempted. One study was predicted to have ‘unclear risk’ because the intention-to-treat (ITT) analysis was not performed properly [41]. Three studies suggested that an imbalance in the number of dropouts could affect the results [37, 38, 42]. However, in two of the three studies, it was unclear whether the reason for the dropout influenced the results and how the ITT analysis responded to the missing data [37, 38].

**Table 2:** Study qualities determined based on the Cochrane Risk of Bias Tool

	Random sequence generation (selection bias)	Allocation concealment (selection bias)	Blinding of participants and personnel (performance bias)	Blinding of outcome assessment (detection bias)	Incomplete outcome data (attrition bias)	Selective reporting (reporting bias)	Other bias
Focht et al., 2017 [39]	+	+	-	+	+	+	+
Foo et al., 2020 [41]	+	+	-	+	?	?	+
Gay et al., 2020 [42]	+	+	-	+	?	+	+
Helminen et al., 2015 [43]	+	+	+	+	+	+	+
Somers et al., 2012 [40]	+	?	-	+	+	?	+
Yip et al., 2007 [37]	+	?	-	?	-	?	+
Yip et al., 2008 [38]	+	?	-	?	-	?	+

(+) low risk of bias, (?) unclear risk of bias, (-) high risk of bias

### Effects of interventions

Effects of interventions are presented in Additional file 3.

#### Self-efficacy for pain

Self-efficacy for pain was measured using the ASES for pain [44] in three studies [37, 38, 42] and Pain Self-Efficacy Questionnaire (PSEQ) [45] in two studies [41, 43]. Self-management education programmes had significant effects on the self-efficacy for pain at 1 month in one study [37], but not in two studies [38, 41]. Self-management education programmes had significant effects on the PSEQ at 6 months [41], but not on ASES for pain at 3 months [42] and 4 months [37, 38]. There was no significant difference between the self-management education and comparator groups at the 12-month follow-up after the intervention [38, 43] .

#### Self-efficacy for other symptoms

Self-efficacy for other symptoms was measured using the ASES for other symptoms [44], such as fatigue, mood, frustration, and physical activity, in three studies [37, 38, 42]. Self-management education programmes had significant effects on self-efficacy for other symptoms at 1 month with moderate effect size [37, 38]. Furthermore, self-management education programmes had significant effects on self-efficacy for other symptoms within 6 months in one study [37], but not in two studies [38, 42]. Nevertheless, self-efficacy for other symptoms still showed a large effect at the 12-month follow-up after the intervention in one study [38].

#### Self-efficacy for function

Self-efficacy for function was measured using the ASES for function [44] in one study [42]. Although the self-efficacy for function was measured at the 3-month follow-up after the intervention, significant differences were not found between the self-management education and comparator groups [42] .

#### Self-efficacy for mobility

Self-efficacy for mobility was measured using the Mobility-Related Self-Efficacy scale that assesses one’s belief in their ability to successfully complete more challenging increments of walking during a 400-m walking task, in one study [39]. Although mobility-related self-efficacy was measured at the 3- and 12-month follow-up after the intervention, significant differences were not found between the self-management education and comparator groups [39] .

#### Self-efficacy for self-regulatory

Focht et al. [39] measured self-regulatory self-efficacy that assesses one’s belief in their ability to successfully organise, plan, and schedule regular exercise and/or physical activity. Self-regulatory self-efficacy was measured at the 3- and 12-month follow-up after the intervention. Self-management education was more effective than the comparator intervention on the self-regulatory self-efficacy measured at both the 3- and 12-month follow-ups [39] .

#### Self-efficacy for weight management

Somers et al. [40] measured the self-efficacy for weight management using the weight efficacy lifestyle questionnaire [46]. Neither the pain coping skill training nor the behavioural weight management had significant effects on the self-efficacy for weight management compared to standard care [40] .

#### Self-efficacy for knee OA

Somers et al. [40] measured the total score of ASES. Pain coping skill training along with behavioural weight management and pain coping skill training alone had significant effects compared to standard care on the ASES total score; however, behavioural weight management alone did not have a significant effect [40] .

## Discussion

This systematic review evaluated the effectiveness of group-based and face-to-face self-management education programmes conducted by health professionals on self-efficacy for patients with knee OA. We included seven articles in this systematic review. Several studies have reported the beneficial effects of self-management education programs on self-efficacy for managing pain and other arthritis symptoms [37, 38, 41] and for self-regulatory [39]. However, the results of the included studies were varied and inconsistent. Additionally, there was a wide range of clinical heterogeneity among these studies regarding sample population, type of intervention, and comparison arms. Therefore, we were unable to validate the results of the included studies, and the effects of group-based and face-to-face self-management education programmes conducted by health professionals on self-efficacy in individuals with knee OA remained inconclusive.

Based on the assessment of the risk of bias, most studies had an ‘unclear’ risk of bias that introduced some weakness in the evidence presented. While random sequence generation was explained clearly in all included studies, most studies categorised ‘high risk’ of bias in terms of blinding of participants. As for the blinding of participants, it may have been difficult to perform blinding due to the nature of the interventions. Other similar systematic reviews also presented weakness scores in blinding of participants [34, 47].

 We included only seven articles, while other similar reviews included more articles. The reason was that the eligibility criteria in this review were more stringent than those in other reviews. We only included studies that included participants having knee OA exclusively based on our research question, while similar studies included people with other arthritis diseases, such as hip OA or RA, inclusively [17–26].

While some studies have reported the short-term and medium-term effects of self-management programmes on self-efficacy for pain management, no studies have reported its long-term effects. In contrast, the effect of intervention programs was observed at 4 weeks and 1 year on self-efficacy for other symptoms, such as frustration, mood, and fatigue. This suggests that self-magement programmes may have a beneficial effect on managing the psychological component. Meanwhile, high-quality studies may be required in the future to develop a strategy to enhance self-efficacy for pain management. At the same time, because OA is a chronic and debilitating condition, self-efficacy for OA may not change for a long time [15]. This may be the reason why the beneficial effects of self-management interventions on self-efficacy does not last for a long time. We must acknowledge the difficulties in altering patients’ behaviour and psychological aspects.

Somers et al. [40] reported that an intervention that included pain coping skill training had a beneficial effect on the ASES total score at 12 months after the intervention in overweight and obese people with knee OA. This suggested that pain coping skill training had a long-term effect on self-efficacy in overweight and obese people with knee OA. However, since this study reported the total score of ASES, it is not clear which sub-item of ASES—pain, function, and other symptoms—was influenced. Besides, the degree to which the intervention affected self-efficacy at each evaluation time point was not clear because the study calculated only post-treatment averages from mixed data measured immediately post-treatment and at 6 and 12 months. An intervention that did not include pain coping skill training but involved only behavioural weight management education was not effective on the ASES total score [40]. The behavioural weight management only intervention could not decrease pain severity. Generally, it is assumed that weight loss is important to reduce pain. However, the results of our review suggest that interventions targeting pain management are required to enhance self-efficacy for pain and reduce pain.

In addition, interventions by Somers et al. [40] had no effect on self-efficacy for weight management. Gay et al. [16] demonstrated in their systematic review that the combination of exercise and education on weight loss was the first-line treatment for hip and knee OA. Therefore, the enhancement of self-efficacy for weight management is important to achieve weight loss in patients with knee OA. However, based on the results of our review, enhancing self-efficacy for weight management may be more challenging than enhancing self-efficacy for pain and arthritis symptoms.

Self-regulatory self-efficacy was improved by an integrated group-based cognitive behavioural counselling with exercise therapy, although mobility-related self-efficacy was not improved (based on the standardised mean difference) [39]. However, cognitive behavioural therapy with exercise resulted in more favourable improvements in mobility-related self-efficacy relative to the comparator group, although the analysis of covariance did not reach conventional levels of significance [39].

Five of the seven studies included in this study had exercise as part of the intervention. Brand et al. [33] reported in their review that exercise interventions used in conjunction with self-management education programmes for individuals with knee OA could not improve self-efficacy more than the self-management education programme alone. Another review also demonstrated that cognitive behavioural therapies, with or without exercise, improved self-efficacy, though it was supported by limited evidence [47]. Conversely, our current review found that most interventions, including exercises, had beneficial effects on self-efficacy [37–40, 42], while two interventions, which included education programmes only, had no beneficial effects on self-efficacy [41, 43]. Therefore, we could not concretely determine whether exercise was required in addition to self-management programmes to improve self-efficacy. However, most clinical guidelines for knee OA recommend exercise as an essential treatment [6, 48, 49]. Therefore, it may still be considered that education programmes with exercise are better than education programmes alone.

Participating in intervention programmes with other participants has been reported to offer more physical and mental benefits than participating alone [29–31]. In general, compared with the Western culture, the Asian culture, including the Japanese culture, emphasises interdependence rather than independence [47]. Group-based programmes are expected to build confidence and increase social interaction and integration into society [8]. Thus, group-based and face-to-face patient education programmes may be better suited for interdependent cultures, such as in Japan.

This current systematic review has some limitations. First, there was a wide range of clinical heterogeneity among the included studies in population, type of intervention, and comparison arms. Included studies in this systematic review were from various countries. There are differences in availability and accessibility of healthcare services between countries, as well as cultural differences resulting in different content of self-management interventions. These differences may also be associated with various comparison arm’s programmes, such as GP care and conventional orthopaedic intervention. Therefore, generalisability and/or applicability of the results of this review should be considered cautiously. Second, we could not perform a meta-analysis with several studies because of the number of included studies (n = 7) and different types of self-efficacy measured (self-efficacy for pain, function, and other symptoms, and mobility, self-regulatory, and weight management self-efficacy). Therefore, we could not synthesise the results of included studies, and the evidence available was inconclusive.

## Conclusions

We reviewed the effects of group-based and face-to-face self-management education programmes conducted by health professionals on self-efficacy in people with knee OA. The current review only included seven studies, and there was a wide range of clinical heterogeneity among the studies. Thus, the effects of group-based and face-to-face self-management education programmes conducted by health professionals on self-efficacy for knee OA exclusively are inconclusive to date. Therefore, high-quality studies are required to provide significant information on clinicians, patients, and healthcare professionals in the future.

## Supplementary Information

**Additional file 1.** Characteristics of the included studies. Presents the total number of participants, inclusion criteria, mean symptom duration, mean age, sex, education, and self-efficacy outcomes of the seven included studies.

**Additional file 2.** Details of the interventions for each of the studies included. Presents the intervention, comparators/controls, subjects, volume, and instructors/facilitators.

**Additional file 3.** Self-efficacy outcome measures used in the included studies. Presents the outcomes, means, SDs, totals of the interventions and controls, and the SMD.

## Data Availability

The datasets used and/or analysed during the current study are available from the corresponding author on reasonable request.

## Abbreviations

ASES: Arthritis Self-Efficacy Scale
CENTRAL: Cochrane Central Register of Controlled Trials
CINAHL: Cumulative Index to Nursing and Allied Health Literature
MeSH: Medical subject headings
OA: Osteoarthritis
OARSI: Osteoarthritis Research Society International
RA: Rheumatoid arthritis
RCT: Randomised controlled trial
PRISMA: Preferred Reporting Items for Systematic Reviews and Meta-Analysis
PSEQ: Pain Self-Efficacy Questionnaire
