# Supplementary material for: Effects of self-management education programmes on self-efficacy for osteoarthritis of the knee: a systematic review of randomised controlled trials
Source: BMC Musculoskelet Disord. 2021 Jun 5;22:515. doi: 10.1186/s12891-021-04399-y (PMC8180097; doi:10.1186/s12891-021-04399-y)
Supplement: Supplementary file 1 — Additional file 1. Characteristics of the included studies. Presents the total number of participants, inclusion criteria, mean symptom duration, mean age, sex, education, and self-efficacy outcomes of the seven included studies. [file 12891_2021_4399_MOESM1_ESM.pdf]

Additional file 1. Characteristics of the included studies

Reference	Country	Total participants (drop out)	Inclusion criteria	Mean symptom duration, years	Mean age, years	Female, %	Education (High school or more), %	Self-efficacy outcomes
Focht et al., 2017. [39]	US	80 (14)	Age 55 + years old, knee pain on most days of the month, Less than 20 min/week of structured exercise during the prior 6 months, Difficulty with basic daily functional tasks due to knee pain, KL 2 or 3 in tibiofemoral OA.	NR	63.5±6.5	IG: 90<br>CG: 78	NR	Self-Regulatory Self-Efficacy, Mobility-Related Self-Efficacy.
Foo et al., 2020. [41]	Malaysia	300 (70)	Age 35-75 years, KL 2 to 4, knee pain on most days for at least a month and rated more than 40/100 on VAS, bony enlargement of the knee.	NR$^{\$}$	NR$^{\$}$	IG: 85.3<br>CG: 80.0	IG: 35.3$^{\#}$<br>CG: 31.3$^{\#}$	Pain Self-Efficacy Questionnaire
Gay et al., 2020. [42]	France	123 (17)	Age 50-75 years, symptomatic knee OA according to the ACR clinical criteria	IG: 12.1±7.7<br>CG: 11.2±7.7	IG: 66.6±6.4<br>CG: 64.7±7.1	IG: 83.3<br>CG: 81.2	NR	ASES for pain, function, and other symptoms
Helminen et al., 2015. [43]	Finland	111 (13)	Age 35-75 years old, KL 2 to 4, knee pain on most days for at least a month and rated more than 40/100 on VAS.	IG: 6.6±4.5<br>CG: 8.9±8.7	IG: 64.5±7.3<br>CG: 62.8±7.2	IG: 71<br>CG: 68	IG: 78<br>CG: 61	Pain Self-Efficacy Questionnaire
Somers et al., 2012. [40]	US	232 (69)	Age > 18 years, knee pain on most days of the month, 25≤BMI≤42, meets the ACR criteria and radiographic evidence of OA affecting one or both knees based on the KL, no other major weight-bearing joint affected by OA, OA of the knee(s) was considered the medical condition that contributed most to limitations in daily function, able to read and speak English.	NR	58.0±10.4	79	98	ASES total score, Weight Efficacy Life-Style Questionnaire.
Yip et al., 2007. [37]	Hong Kong	182 (62)	Meets the ACR clinical criteria*	8	65	75	NR	ASES for pain and other symptoms.

Yip et al., 2008. [38]	Hong Kong	95 (42)	Meets the ACR clinical criteria*	IG: 8.0±6.0<br>CG: 6.7±6.0	IG: 64.8±10.6<br>CG: 63.4±10.7	IG: 89<br>CG: 82	IG: 11.1<br>CG: 14.0	ASES for pain and other symptoms.

ACR; American College of Rheumatology, ASES; Arthritis Self-efficacy Scale, CG; Control group, IG; Intervention group, KL; Kellgren -Lawrence grade, NR; Not recorded, OA; Osteoarthritis, US; United States, VAS; Visual Analog Scale. *The clinical criteria for knee OA consisted of pain in the knee and any three of the following: (1) aged ≥ 50 years; (2) <30 minutes of morning stiffness; (3) crepitus on active motion; (4) bony tenderness; (5) bony enlargement; or (6) no palpable joint warmth. $Symptom duration and age were described as frequency in the related article (Mal J Med Health Sci 13(2). 7-15. 2017.). #Tertiary education (aged ≥18 years).
