# Supplementary material for: Effects of self-management education programmes on self-efficacy for osteoarthritis of the knee: a systematic review of randomised controlled trials
Source: BMC Musculoskelet Disord. 2021 Jun 5;22:515. doi: 10.1186/s12891-021-04399-y (PMC8180097; doi:10.1186/s12891-021-04399-y)
Supplement: Supplementary file 2 — Additional file 2. Details of the interventions for each of the studies included. Presents the intervention, comparators/controls, subjects, volume, and instructors/facilitators. [file 12891_2021_4399_MOESM2_ESM.pdf]

Additional file 2. Details of the interventions for each of the studies included

Reference	Intervention	Comparator/ Control	Subjects, n	Volume	Instructors/ facilitators	Follow-up
Focht et al., 2017. [40]	20 min of group-based cognitive behavioural counselling + 60 min of exercise	60 min of exercise	NR	IG: One session/wk to /mo for 36 wks (total 27 sessions, 36 contact hours). CG: Three sessions/wk for 12 wks.	NR (study stuff)	3 mos 12 mos
Foo, et al. 2020. [41]	2.5 hrs of cognitive-behavioural therapy + standard routine care + education booklet	Standard routine care + education booklet	8-12	IG: one session/2 wks for 6 wks	PT and nurse	1 mo 6 mos
Gay, et al. 2020. [43]	90 min of self-management exercise education including PA practice + spa therapy + education booklet	Spa therapy + education booklet	5-7	IG: 18 spa therapy sessions during 3 wks + 5 self-management exercise sessions during 16 days CG: 18 spa therapy sessions during 3 wks	IG: PT, PA instructor CG: PT	3 mos
Helminen et al., 2015. [44]	2 hrs of Cognitive-behavioural training for pain management + ordinary GP care	Ordinary GP care	7-13	IG: One session/wk for 6 wks.	IG: Psychologist, PT CG: GP	3 mos 12 mos
Somers et al., 2012. [42]	PCST: 1 hr of pain coping skill training program.	Standard care	6-10	PCST: one session/wk for the	PCST: Psychologist BWM: Psychologist,	6 mos 12 mos

	or<br>BWM: 1 hr group session of behavioural weight management + 1.5 hrs supervised exercises session<br>or<br>PCST + BWM			first 12 wks and /2wks for the last 12 wks.<br>BWM: one group session and three exercise sessions/wk for the first 12 wks and one group session/2wks for the last 12 wks.	Exercise physiologist	
Yip et al., 2007. [38]	2 hrs of modified Arthritis Self-management program including exercise + conventional orthopaedic treatment	Conventional orthopaedic treatment	10-15	IG: One session/wk for 6 wks	Registered nurse	1 wk<br>16 wks
Yip et al., 2008. [39]	2 hrs of modified Arthritis Self-management program including exercise +conventional orthopaedic treatment	Conventional orthopaedic treatment	10-15	IG: One session/wk for 6 wks	Registered nurse	1 wk<br>16 wks<br>1 yr

BWM; Behavioral weight management, CG; Control group, GP; General Practitioner, IG; Intervention group, PCST; Pain coping skill training, PA; Physical activity, PT; Physiotherapist
