# Supplementary material for: Effects of self-management education programmes on self-efficacy for osteoarthritis of the knee: a systematic review of randomised controlled trials
Source: BMC Musculoskelet Disord. 2021 Jun 5;22:515. doi: 10.1186/s12891-021-04399-y (PMC8180097; doi:10.1186/s12891-021-04399-y)
Supplement: Supplementary file 3 — Additional file 3. Self-efficacy outcome measures used in the included studies. Presents the outcomes, means, SDs, totals of the interventions and controls, and the SMD. [file 12891_2021_4399_MOESM3_ESM.pdf]

Additional file 3. Summary of results in included studies

Outcome	Intervention Mean	Intervention SD	Intervention Total	Control Mean	Control SD	Control Total	SMD (95% CI)
Self-efficacy for pain at 1 month							
ASES for pain [37]	34.89	6.27	88	32.75	7.80	94	0.30 (0.01, 0.59)
ASES for pain [38]	35.16	6.17	40	34.09	7.01	37	0.16 (-0.29, 0.61)
PSEQ [41]	3.38	1.16	126	3.18	1.08	128	0.18 (-0.17, 0.42)
Self-efficacy for pain within 6 months							
ASES for pain, 4 months [37]	36.09	13.09	88	33.27	7.98	94	0.26 (-0.03, 0.55)
ASES for pain, 4 months [38]	36.83	6.23	40	35.17	7.71	37	0.24 (-0.21, 0.68)
PSEQ, 6 months [41]	3.71	1.24	119	3.35	1.43	111	0.27 (0.01, 0.53)
ASES for pain, 3 months [42]	6.50	1.70	52	6.00	1.80	54	0.28 (-0.10, 0.67)
Self-efficacy for pain at 12 months							
ASES for pain [38]	38.30	7.02	40	35.48	7.46	37	0.39 (-0.07, 0.84)
PSEQ[*1] [43]	43.10	11.10	55	46.20	10.82	56	-0.28 (-0.65, 0.09)
Self-efficacy for other symptoms at 1 month							
ASES for other symptoms [37]	43.61	6.63	88	40.09	8.57	94	0.46 (0.16, 0.75)
ASES for other symptoms [38]	43.56	6.59	40	39.86	8.18	37	0.50 (0.04, 0.95)
Self-efficacy for other symptoms within 6 months							
ASES for other symptoms, 4 months [37]	42.92	8.44	88	40.12	9.09	94	0.32 (0.02, 0.61)
ASES for other symptoms, 4 months [38]	45.21	7.56	40	41.68	8.56	37	0.43 (-0.02, 0.89)
ASES for other symptoms, 3 months [42]	7.20	1.80	52	6.90	1.90	54	0.16 (-0.22, 0.54)
Self-efficacy for other symptoms at 12 months							
ASES for other symptoms, 12 months [38]	46.75	8.15	40	41.03	8.90	37	0.66 (0.20, 1.12)
Self-efficacy for function							
ASES for function, 3 months [42]	7.70	1.90	52	7.60	1.70	54	0.06 (-0.33, 0.44)
Self-efficacy for mobility							
MRSE, 3 months [39]	81.42	26.28	40	74.17	27.05	40	0.27 (-0.17, 0.71)

MRSE, 12 months [39]	81.54	27.10	40	71.63	28.25	40	0.35 (-0.09, 0.80)
Self-efficacy for self-regulatory							
SRSE, 3 months [39]	63.50	18.77	40	52.50	19.98	40	0.56 (0.11, 1.01)
SRSE, 12 months [39]	62.25	16.09	40	46.94	22.50	40	0.78 (0.32, 1.23)
Self-efficacy for weight management							
WELSQ*2, 12 months, vs PCST+BWM [40]	6.50	3.20	62	5.70	1.10	51	0.33 (-0.05, 0.70)
WELSQ*2, 12 months, vs BWM [40]	5.90	1.15	59	5.70	1.10	51	0.18 (-0.20, 0.55)
WELSQ*2, 12 months, vs PCST [40]	6.00	1.16	60	5.70	1.10	51	0.27 (-0.11, 0.64)
Self-efficacy for knee OA							
ASES total score*2, 12 months, vs PCST+BWM [40]	243.25	27.37	62	213.00	29.51	51	1.06 (0.66, 1.46)
ASES total score*2, 12 months, vs BWM [40]	222.30	29.16	59	213.00	29.51	51	0.31 (-0.06, 0.69)
ASES total score*2, 12 months, vs PCST [40]	225.70	30.97	60	213.00	29.51	51	0.42 (0.04, 0.79)

*1The scores calculated as mixed data from 3 and 12 months.

*2The scores calculated as mixed data from post-treatment, 6 months, and 12 months.

ASES; Arthritis Self-Efficacy Scale, BWM; Body weight management, CI; Confidence interval, MRSE; Mobility-Related Self-Efficacy, PCST; Pain Coping Skill Training, PSEQ; Pain Self-Efficacy Questionnaire, SD; Standard deviation, SMD; Standardised mean difference, SRSE; Self-Regulatory Self-Efficacy,

WELSQ; Weight Efficacy Life-Style Questionnaire,
